# Health literacy assessment and analysis of influencing factors in pregnant women with gestational diabetes mellitus in Southwest China

**DOI:** 10.3389/fpubh.2024.1477706

**Published:** 2025-01-07

**Authors:** Fangmei Tang, Li Gu, Xiujing Guo, Wenjing Fu, Benyi He, Yuqing Song, Dehua Li

**Affiliations:** ^1^Department of Obstetric Nursing, West China Second University Hospital, Sichuan University, Chengdu, China; ^2^Key Laboratory of Birth Defects and Related Diseases of Women and Children (Sichuan University), Ministry of Education, Chengdu, China; ^3^Department of Gynecological Nursing, West China Second University Hospital, Sichuan University, Chengdu, China; ^4^Department of Hepatobiliary Pancreatic Vascular Surgery, The First Hospital of Kunming, Kunming, China; ^5^Office of Operations Management and Evaluation, West China Second University Hospital, Sichuan University, Chengdu, China

**Keywords:** health literacy, gestational diabetes mellitus, GDM, social support, self-efficacy, HLS-14

## Abstract

**Objective:**

The main objectives of our study are evaluating the health literacy level among women with gestational diabetes mellitus (GDM) in Southwest China and explore the influencing factors, using a multidimensional health literacy assessment scale (Chinese version of the HLS-14). Given that the HLS-14 has not been used in GDM previously, its reliability and validity testing was included as a secondary objective.

**Method:**

It was a cross-sectional survey with 565 GDM pregnancies. The Maternal and child health information access questionnaire, Chinese version of the HLS-14, Perceived Social Support Scale (PSSS) and General Self-efficacy Scale (GSES) was used to collect health information access behaviors, health literacy, social support and self-efficacy levels, respectively. SPSS 21.0 was used for descriptive statistical, multiple stepwise linear regression analysis and exploratory factor analysis (EFA). Amos 26.0 was used for confirmatory factor analysis (CFA).

**Results:**

The Chinese version of HLS-14 has good reliability and validity in GDM pregnancies. The Cronbach’s α are 0.849, 0.866, 0.859, and 0.883, respectively. The exploratory factor analysis extracted three common factors with a cumulative variance contribution rate of 68.405%. The confirmatory factor analysis model fit was good (χ^2^/df = 2.595, RMSEA = 0.055, IFI = 0.970, TLI = 0.963, CFI = 0.970). The HL level in pregnancies with GDM was moderate with a mean score of 3.26 ± 0.41, of which 24.10% had limited HL, 41.87% had moderate HL, and 34.03% had adequate HL. Regression analysis showed that the women with higher family support (*β* = 0.298, *p* < 0.001), recording pregnancy management diary (*β* = 0.199, *p* < 0.001), higher the family income (β = 0.140, *p* < 0.001), lower pre-pregnancy BMI (*β* = −0.116, *p* = 0.004), longer time spent searching for health information (β = 0.111, *p* = 0.006), and searching for health information through a medical health information website (β = 0.093, *p* = 0.019) had higher HL levels. These variables explained 23.1% of the variance in HL.

**Conclusion:**

The Chinese version of the HLS-14 has good applicability in the GDM pregnancies. The HL level of them is moderate, needs to be improved. Healthcare professionals should focus on the GDM population with low family income and high pre-pregnancy BMI, fully mobilize their social support system, provide reliable access to information, encourage all GDM pregnancies to use pregnancy management diaries to record their self-management behaviors, and ensure the effectiveness of health education.

## 1 Introduction

Gestational Diabetes Mellitus is an abnormality of glucose metabolism that is first detected at any time during pregnancy but does not meet the diagnostic criteria for non-pregnant overt diabetes mellitus, is usually detected by routine testing between the 24th and 28th weeks of gestation, and is one of the most common pregnancy comorbidities that can lead to serious adverse pregnancy outcome (1). With its high prevalence and high disease burden, GDM is a global public health problem. The trend of its prevalence is on the rise, and according to the International Diabetes Federation estimates that 15.8% (20.4 million) of live births in 2019 were affected by hyperglycemia in pregnancy, of which 85.1% were GDM, and the great majority of cases occurred in low- and middle-income countries (2); A systematic review involving 79,064 Chinese showed that the incidence of GDM in mainland China was 14.8% (3); In China, the annual economic burden of GDM to society is about 19.36 billion yuan, and the average medical expenditure of each GDM pregnant woman is 6677.37 yuan more than normal pregnancies (4). The etiology of GDM is unclear, but is affected by a range of risk factors including sociodemographic factors as well as physiologic factors, such as family history of diabetes, advanced age, overweight/obesity, poor lifestyle, and polycystic ovary syndrome. GDM poses a serious threat to the physical and mental health of mothers and infants (5, 6). On the one hand, GDM may cause serious immediate and long-term adverse effects on the health of mother and child (5, 7): such as increased risk of preeclampsia, preterm labor, cesarean section, shoulder dystocia in the perinatal period, and insufficient lactation in mothers; increased risk of long-term diseases, including type 2 diabetes mellitus, metabolic syndrome, cardiovascular disease, etc. The risk of fetal macrosomia, neonatal hypoglycemia, hyperbilirubinemia, congenital malformations, and admission to the neonatal intensive care unit is increased; the future risk of diabetes mellitus, metabolic syndrome, overweight and obesity, insulin resistance, and cardiovascular disease is also increased; On the other hand, the mental health is also threatened. Studies have shown that the experience of GDM increased maternal psychological burden and emotional damage, leading to mental health problems such as anxiety and depression (8).

The management of GDM is closely dependent on maternal self-management behaviors, which are influenced by a number of factors, including demographic and psychosocial factors. It has been found that the risk of poor glycemic control is considerably increased in GDM pregnant women with inadequate health literacy, which indicated that health literacy may be an important predictor of inadequate self-management behaviors in GDM pregnant women (9, 10). Health literacy is “people’s motivation, knowledge, and competence to access, understand, appraise, and apply health information in order to make judgments and decisions about health care, disease prevention, and health promotion in their daily lives, in order to maintain or improve the quality of life over the course of the life course” (11). The improvement of the public’s HL is recognized as one of the goals of the national health strategic plan in many countries (12). Health literacy is a multidimensional concept, functional HL is considered to be basic skills that are necessary in health settings, such as the ability to read and write; whereas communicative and critical HL are considered to be more advanced cognitive skills that allow pregnant women with GDM to comprehend, analyze, and apply health information, and take appropriate health self-management behaviors (13). There is a closely relationship between HL and health behaviors, which is a prerequisite and guarantee for the realization of health behaviors, prompting patients to adopt correct and effective self-management behaviors, and the implementation of health behaviors can also further improve HL levels and form good health habits and beliefs. Previous studies have confirmed that HL affects self-management behaviors of type 2 diabetes patients. A study found that HL was a mediator of the relationship between formal education and glycemic control through a path analysis comparing HL and formal education among approximately 400 low-income diabetic patients (14). It has also been reported that higher diabetes knowledge scores are associated with better functional, communicative, and critical HL, and people with adequate disease knowledge may feel more confident and comfortable when communicating with healthcare professionals (15, 16). It was also shown that although HL does not have a direct effect on glycemic control, an indirect effect works through diabetes knowledge (17). HL might influence glycemic control and self-management behaviors of patients with type 2 diabetes mellitus as well, both through a direct effect and an indirect effect mediated by self-efficacy (18, 19).

An adequate access to, understanding of, and application of health information is important, and this is especially prominent during pregnancy, when behaviors can affect maternal and fetal health. HL may be an important factor influencing self-management behaviors and pregnancy-related outcomes in pregnant women with GDM. During pregnancy, pregnant women are confronted with a wide range of health information from different sources that contain advice on healthy behaviors. The studies have shown that pregnant women with insufficient HL exhibit poorer adherence to folic acid intake and regular obstetric checkups, longer hospitalization, and shorter periods of exclusive breastfeeding during their pregnancies despite clear evidence-based advice and health materials from medical professionals (20–22); insufficient HL make it more difficult to access and understand information about pregnancy and prenatal checkups, which can be detrimental to making informed medical decisions (23, 24). In addition, women with insufficient HL also have more negative beliefs about medicines (25), and show more anxiety about the results of labor and delivery tests (26); HL has been suggested to be an important factor indirectly influences pregnancy outcomes, pregnant women with high HL levels more likely to have a better pregnancy outcome (27), which is most likely due to the fact that HL has a great direct or indirect impact on self-management behaviors.

Investigating the level of HL and the factors influencing it among pregnant women with GDM is beneficial for health care providers to develop effective interventions to improve their HL, which in turn improves their self-management behaviors. However, little is known about the HL level of the GDM population currently. There is also a lack of a multidimensional, rapid measurement tool to assess HL in pregnant women with GDM. Choosing an appropriate multidimensional HL assessment tool is also very crucial to understand the real HL level of them. There are more existing HL assessment tools, including universal scales and scales for specific diseases or special populations. Early health literacy assessment tools, primarily used in healthcare settings, focused on assessing patients’ functional HL in reading, numeracy, and comprehension in order to quickly screen for health literacy deficiencies. These include the rapid evaluation of adult literacy in medicine (REALM) (28), The test of functional health literacy in adults (TOFHLA) (29), The newest vital sign (NVS) (30), and the Brief Health Literacy Questionnaire (BHLS) (31); However, with the continuous enrichment and development of the concept of HL, its assessment has gradually focused on the comprehensive evaluation of an individual’s ability to access, understand, appraise, and apply health information. Among the multidimensional HL measurement scales, relatively representative scales include the Health Literacy Scale (HLS-14) (15), the European Health Literacy Questionnaire (HLS-EU-Q) (32), the Health Literacy Scale for Chronic Diseases (HELMS) (33), and the Health Literacy Questionnaire (HLQ) in Australia (12). Among them, HLS-14 is developed by Japanese scholars Ishikawa, based on the theoretical basis of Nutbeam’s classification of the 3 levels of HL, which includes 14 entries in 3 dimensions, including functional HL, communicative HL, and critical HL, and it can comprehensively assess the HL of diabetic patients in these 3 dimensions. It has been translated into different languages and has been widely used in many countries (Japan, Germany, Korea, Netherlands, the United States, and French), and has been widely used in different populations (adults, chronically ill older adults, breast cancer, rheumatism, diabetic patients, etc.) (18, 34–38). Compared with assessment instruments focusing on functional HL such as literacy, its test scores are normally distributed without a ceiling effect, should be highly recommended when assessing the HL of people with higher educational level (39). Chinese scholars Xiaoyan Zhao translated and culturally adapted it to form the Chinese version of the HLS-14 (40), which has a good reliability and validity, and has been used to measure HL in Chinese patients with type 2 diabetes, but has not yet been used in the GDM.

The main objectives of our study are to assess the health literacy level of pregnant women with GDM in Southwest China by a multidimensional health literacy assessment scale (Chinese version of the HLS-14) and to explore the influencing factors. The HLS-14 is a well-established scale, but the Chinese version of the HLS-14 has been previously applied only in the type 2 diabetes mellitus. Considering the differences in the investigate groups, we also assess the reliability and validity of the HLS-14 in our participants before the formal investigation.

## 2 Materials and methods

### 2.1 Participants and data collection procedure

This study was a cross-sectional survey study. All subjects met the following criteria: (i) Initial diagnosis of GDM during pregnancy and met the diagnostic criteria for GDM of the IADPSG 2010 (41); (ii) Age ≥ 18 years; (iii) Those who have an elementary understanding of reading and no communication disabilities; and gestational weeks ≥28 weeks. Pregnant women with GDM who had pre-pregnancy diabetes, multiple pregnancies, and combined severe medical, surgical or obstetric complications were excluded. Using a convenience sampling method, women with GDM who underwent obstetric examination or were hospitalized in the West China Second University Hospital, Sichuan University between December 2021 and June 2022 were selected. The study was approved by the Medical Ethics Committee of the West China Second University Hospital, Sichuan University (No. 2021-219), and verbal informed consent was obtained from each of the participants.

All the questionnaires were distributed by our team members after obtaining the consent of the pregnant women, and they were instructed to fill them out. A total of 620 pregnant women with GDM were invited to participate in the study, with 565 agreeing and 55 refusing to participate. Questionnaires with greater than 10% missing items or greater than 50% missing items on any subscale were treated as invalid. Missing values for scales were filled using multiple interpolation method, and missing data for demographic variables are not filled in.

### 2.2 Theoretical framework

Integrated model of health literacy is a new health literacy model proposed by Sørensen K in 2012 based on literature reviews and expert opinions, synthesizing 17 previous definitions of HL and 12 pre-existing conceptual models for content analysis, in which a team of experts from the European Commission on Health Literacy participated (11). This model combines the qualities of a conceptual model and a logistic model, with the conceptual model outlining the 12 dimensions that HL encompasses, which refer to the knowledge, motivation, and ability to access, understand, evaluate, and apply health-related information in healthcare, disease prevention, and health promotion settings, respectively. The logic model shows the individual- and system-level factors that influence HL and the pathways that link HL to health outcomes. According to the model’s connotation, the factors affecting individual HL mainly include personal characteristics, environmental characteristics, and socio-environmental factors.

Among the factors influencing HL, there is a distinction between distal factors, which include social and environmental determinants (e.g., demographic status, culture, language, political power, social systems), and proximal factors, which pay more attention to individual determinants (e.g., age, gender, ethnicity, socio-economic status, education, occupation, employment, income, literacy) and situational determinants (e.g., social support, the influence of family and peers, media use, and physical environment). Based on the literature review, and according to the proximal and distal factors affecting HL in the conceptual model, we included the possible influencing factors of HL in pregnant women with GDM, including: (1) personal characteristics (personal determinants): such as ethnicity, occupation, literacy level, economic conditions, pregnancy and childbirth history, family history of diabetes mellitus, personality type, and self-efficacy, etc.; (2) environmental characteristics (situational determinants): place of residence, social support, marital status, husband-wife relationship, access to health information, and health education, etc.; and (3) socio-environmental factors: the way of medical payment.

### 2.3 Measures

Demographic Characteristics Form: Basic demographic information included in age, education level, income level, marital status, occupation, parity, length of pregnancy, family history of diabetes, and so on.

Maternal and child health information acquisition questionnaire: including health information-seeking behavior, channels for acquiring information, and evaluation of information.

Health Literacy Scale (HLS-14): This is a multidimensional health literacy scale developed by Japanese scholars Ishikawa (15), based on Nutbean’s Health Literacy Model (42). It consists of 14 items 3 dimensions: functional HL, communicative HL, and critical HL. The Chinese version was translated and culturally adapted by Zhao et al. (40). It’s scored on a four-point Likert scale, with each item rated on a scale of 1–4 from “never” to “often” (functional HL dimension is reverse scored). The final result is typically expressed as the mean score of the 14 items. The Cronbach’s *α* was 0.853 in our study.

Perceived Social Support Scale (PSSS): This is a widely used scale to measure social support, developed by Zimet and revised by Zhong et al. (43). The scale has 12 items and is divided into three dimensions: family support, friend support and other support. Each item was rated from 1 to 7 on a 7-point Likert scale from “strongly disagree” to “strongly agree,” with a score range of 7–84. The final result is the sum of the scores of all items, with higher total score indicating stronger social support. The Cronbach’s α was 0.953 in our study.

General Self-efficacy Scale (GSES): General self-efficacy measures, to some extent, the confidence of an individual in the face of a variety of unfamiliar environments or encountering new things, and helps people to develop a comprehensive and in-depth understanding and achieve good results. German scholars Schwarzer and his colleagues developed this scale in 1981, which contains 10 items on a 4-point Likert scale, with each item scoring from 1 to 4 on a scale from “not at all correct” to “completely correct” (44). The Cronbach’s *α* was 0.930 in our study.

### 2.4 Statistics

Data were entered using Excel 2019, SPSS 21.0 software for regression analysis, exploratory factor analysis, and Amos 26.0 software for validation factor analysis of HLS-14.

Reliability and validity: reliability was analyzed using retest reliability, internal consistency (Cronbach’s α coefficient); EFA and CFA were used to measure the structural validity of the scales. The maximum likelihood method was selected for model parameter estimation, and the model fitness indexes were selected and evaluated (45): Relative Chi-Square/DF, (χ^2^/df) < 3.0; Root Mean Square Error of Approximation, (RMSEA) < 0.08; Incremental Fit Index (IFI), Tucker-Lewis index (TLI), and Comparative Fit Index (CFI) were all > 0.9.

## 3 Results

### 3.1 Demographic characteristics and pregnancy-related conditions

A total of 565 pregnant women with GDM were surveyed in this study, and a total of 523 valid questionnaires were obtained, excluding 42 questionnaires that were not properly or incompletely completed. The average age of the participants was 32.11 ± 3.92 years old, the average length of pregnancy was 35.36 ± 2.91 weeks, 97.9% were Han Chinese, 94.2% lived in urban areas, 98.7% were married, 71.3% had a bachelor’s degree or above, 87.6% were employed, 89.5% had social security, 66.5% had a per capita monthly household income >8,000 RMB (equivalent to approximately US$1,160). 68.6% were pregnant with their first child; 93.9% did not use insulin for blood glucose control, and 75.1% had no family history of diabetes. 85.66% had received GDM health education from medical personnel during pregnancy, and only 19.89% had received GDM health education in community settings; 47.23% had relatives or friends who were engaged in healthcare-related work; 90.25% self-reported their personality type as extroverted or between introverted and extroverted, and 86.62% had a good relationship with their partners; most of the pregnant women adhered to keep (49.71%) or occasionally kept (43.79%) a pregnancy management diary to manage their pregnancy diet, exercise, etc. Pregnant women with GDM had a PSSS score of (65.06 ± 11.40) with a high level of social support received, and a GSES score of (26.64 ± 5.34) with a moderate level of general self-efficacy.

### 3.2 Applicability analysis of the Chinese version of HLS-14 in GDM pregnant women

#### 3.2.1 Expert consultation and semantic adaptation

Prior to the formal survey, we conducted an expert consultation on the HLS-14 guidelines and the content of the entries with two clinical GDM nursing experts, who had no comments on the scale entries and suggested that some of the scale guidelines should be changed. After correcting the guidelines according to the experts’ opinions, we distributed the scale to 30 pregnant women with GDM to fill in and asked for feedback, and all 30 pregnant women with GDM indicated that the scale was clear and easy to understand, and there were no ambiguities or difficult-to-understand expressions. The average time to complete the scale was 1.5 min.

#### 3.2.2 Validity and reliability analysis

Analysis of retest reliability and internal consistency. Re-test reliability was assessed using the correlation coefficient between the scores of the two repeated measures of the HLS-14, and the interval between repeated measures ranged from 10 to 14 days; a total of 14 pregnant women with GDM completed the retest questionnaire. The results are shown in Table 1.

**Table 1:** The results of the re-test reliability and internal consistency test for each dimension of HLS-14.

Items	Re-test reliability (*n* = 14)	Cronbach’s α (*n* = 149)
HLS-14	0.885**	0.849
Functional HL	0.768**	0.866
Communicative HL	0.876**	0.859
Critical HL	0.695*	0.883

*p < 0.05, **p < 0.01.

Exploratory factor analysis: EFA was conducted using the questionnaire samples (*n* = 149) collected during the pre-project period. The results showed that the KMO of the HLS-14 was 0.834, and the approximate chi-square value of the Bartlett’s test of sphericity was 1194.763 (*p* < 0.001), indicating that the individual entries of the scale have a good correlation with each other, and that a factor analysis can be conducted. Principal component analysis was performed using the maximum variance orthogonal rotation method, and the common factors were extracted according to the principle of eigen root >1. The results showed (Tables 2, 3) that three common factors were extracted for the 14 entries, with a cumulative variance contribution rate of 68.405%, and the loadings of each measurement question item on the corresponding factor were all >0.50, and the common degree of each entry was all >0.5, with no spanning factor, and the results of the dimensional divisions were consistent with the original scale. It indicates that the structure of the Chinese version of the HLS is stable.

**Table 2:** Factor analysis of the loading values (*n* = 149).

Items	Loading values			Communality
	Factor 1	Factor 2	Factor 3	
HLS 3	0.893			0.759
HLS 4	0.853			0.676
HLS 2	0.848			0.720
HLS 1	0.759			0.558
HLS 5	0.621			0.506
HLS 8		0.851		0.771
HLS 9		0.794		0.660
HLS 10		0.791		0.730
HLS 7		0.784		0.667
HLS 6		0.535		0.549
HLS 12			0.862	0.831
HLS 11			0.848	0.721
HLS 13			0.833	0.790
HLS 14			0.722	0.745

Factor 1 = functional HL, factor 2 = communicative HL dimension, and factor 3 = critical HL.

**Table 3:** Total variation explained by the HLS-14 extraction factor (*n* = 149).

	Factor 1	Factor 2	Factor 3
Latent root	5.085	3.075	1.417
Contribution rate (%)	23.360	22.670	22.375
Accumulative contribution rate (%)	23.360	46.030	68.405

**Figure 1:**
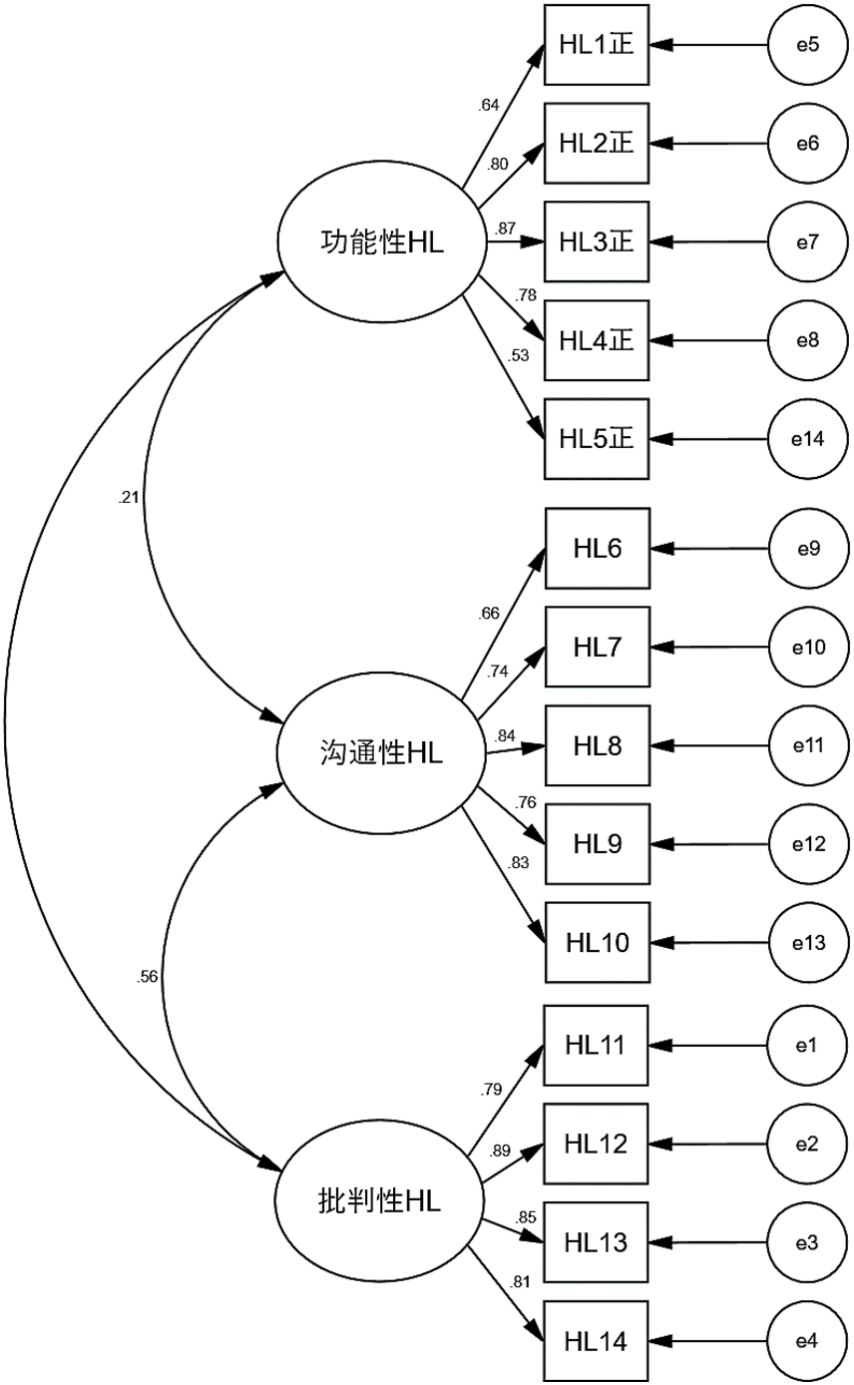
The validation factor analysis model diagrams of the HLS-14. 功能性HL, functional health literacy; 沟通性HL, communicative health literacy; 批判性HL, critical health literacy.

Confirmatory factor analysis: We found that the HLS-14 has good retest reliability in the GDM population, and exploratory factor analyzes confirmed that the scale has good construct validity. Following this foundation, we conducted further explorations. The CFA for HLS-14 was performed using a sample collected at follow-up (*n* = 374 cases). The three scale dimensions extracted from the EFA were included in the structural equation modeling as latent variables, and the measurement entries corresponding to each dimension were included in as measurement variables to test model fitness, the results showed that χ^2^/df = 2.595, within the excellent range of 1 ~ 3; RMSEA = 0.055, within the good range of <0.08; IFI, TLI, and CFI were all >0.9, reaching the excellent level, which indicated that the Chinese version of the three-factor CFA model for HLS had good fitness. The results of the validation factor analysis are shown in Figure 1.

### 3.3 Analysis of the status and influencing factors of health literacy in pregnant women with GDM

#### 3.3.1 The status of health literacy in pregnant women with GDM

The results showed that the total mean HL score for women with GDM was 3.26 ± 0.41. The mean scores for the functional, communicative, and critical HL dimensions were 3.19 ± 0.61, 3.32 ± 0.56, and 3.27 ± 0.6. The three items with the highest scores were “Needs help with reading “in the functional HL dimension, “Can apply the information obtained in daily life” and “Can understand the information obtained” in the communicative HL dimension. The three items with the lowest scores were “Can understand the information obtained.” The three lowest scoring items were all in the Functional HL dimension: “Takes a long time to read and understand “, “Find the font too small “, and “Find the content too difficult to understand” (Table 4).

**Table 4:** The score of HL-14 in pregnant women with GDM (*n* = 523).

Items	^−^x ± s	Scoring range	Dimension
Needs help with reading	3.65 ± 0.59	1 ~ 4	Functional HL
Can apply the information obtained in daily life	3.42 ± 0.62	1 ~ 4	Communicative HL
Can understand the information obtained	3.40 ± 0.66	1 ~ 4	Communicative HL
Considered whether the information applies to your situation	3.31 ± 0.64	1 ~ 4	Critical HL
Collect information and make decisions about your health	3.30 ± 0.67	1 ~ 4	Critical HL
Can share your ideas about health with others	3.30 ± 0.70	1 ~ 4	Communicative HL
Considers whether the information is correct	3.27 ± 0.70	1 ~ 4	Critical HL
Can collect information from a variety of sources	3.24 ± 0.75	1 ~ 4	Communicative HL
Can find the information you want quickly	3.23 ± 0.69	1 ~ 4	Communicative HL
Check that the information is correct	3.23 ± 0.72	1 ~ 4	Critical HL
Finding words or phrases that you do not know or do not understand	3.14 ± 0.79	1 ~ 4	Functional HL
Find the content too difficult to understand	3.08 ± 0.76	1 ~ 4	Functional HL
Find the font too small	3.04 ± 0.79	1 ~ 4	Functional HL
Takes a long time to read and understand	3.02 ± 0.79	1 ~ 4	Functional HL

According to the criteria for determining HL levels in previous studies (46, 47), the HL levels of pregnant women with GDM in this study were as follows (Table 5).

**Table 5:** Distribution of HL levels in pregnant women with GDM (*n* = 523).

Dimension	Limited HL		Moderate HL		Adequate HL	
	Number	Ratio (%)	Number	Ratio (%)	Number	Ratio (%)
Overall HL	126	24.10	219	41.87	178	34.03
Functional HL	156	29.83	200	38.24	167	31.93
Communicative HL	76	14.53	226	43.21	221	42.26
Critical HL	88	16.83	217	41.49	218	41.68

Criteria for the determination of HL: A mean score of <3 on the HL-14 or each dimension was considered limited HL, ≥3 and <3.5 was considered moderate HL, and ≥3.5 was considered adequate HL.

#### 3.3.2 The analysis of factors influencing the HL of pregnant women with GDM

Before the multifactor analysis, we conducted a univariate analysis of the variables. Then the mean score of the HL-14 was used as the dependent variable, and the variables with significance in the univariate analysis (*p* < 0.05) were used as independent variables. The stepwise regression method was used to enter the multiple linear regression model. The existence of covariance between independent variables was judged based on Tolerance and Variance Inflation Factor (VIF), and the existence of autocorrelation of variables was judged based on Durbin-Watson value. The tolerance is >0.1, VIF is between 1.021 and 1.080, and Durbin-Watson value is 2.046, which indicates that there is no covariance and autocorrelation among the variables in this multiple linear regression equation.

The results showed that family support, use of a pregnancy management diary, family monthly income, pre-pregnancy BMI, time spent searching for health information, and searching for health information through medical information websites entered the model of factors influencing the HL of pregnant women with GDM (*F* = 26.114, *p* < 0.001), with an adjusted R2 of 0.231, i.e., it could explain 23.1% of the total variation in the HL of pregnant women with GDM (Table 6).

**Table 6:** Multiple stepwise linear regression analysis of factors influencing HL in pregnant women with GDM (*N* = 523).

Variables	Non-standardized coefficient		Standardized coefficient	*t*	*p*	Covariance	
	*B*	SE	β			Tolerance	VIF
(Constant)	2.299	0.157		14.599	<0.001		
Family support	0.116	0.016	0.298	7.433	<0.001	0.953	1.049
Pregnancy management diary	0.131	0.026	0.199	4.952	<0.001	0.950	1.052
Family income	0.030	0.009	0.140	3.431	<0.001	0.926	1.080
Pre-pregnancy BMI	−0.015	0.005	−0.116	−2.899	0.004	0.965	1.036
Time spent searching for health information	0.053	0.019	0.111	2.763	0.006	0.942	1.061
Searching for health information through medical information websites	0.084	0.036	0.093	2.360	0.019	0.979	1.021

*R2 = 0.240, adjusted R2 = 0.231, F = 26.114, p < 0.001.*

## 4 Discussion

### 4.1 Applicability of the Chinese version of HLS-14 in pregnant women with GDM

#### 4.1.1 Reliability and validity of the HL-14

Reliability is used to evaluate whether a scale or questionnaire can obtain consistent, trustworthy as well as stable results, and its basic characteristics include stability, homogeneity and equivalence. In this study, the stability and homogeneity of the Chinese version of the HLS-14 were evaluated by retest reliability and Cronbach’s *α* coefficient. The results showed that the Chinese version of the HLS-14 showed good stability and internal consistency, similar to the scale’s measurements in diseases such as type 2 diabetes mellitus and end-stage renal disease (40, 47, 48), as well as in the general population (34, 38).

Validity is the validity or correctness of the measurement results of a tool. This study used exploratory factor analysis and validation factor analysis to measure the construct validity of the scale. Similar to previous studies (15, 38, 40), the exploratory factor analysis in this study extracted a total of three male factors with a cumulative variance contribution of 68.405%, and each item loaded >0.50 on the corresponding factor, the scale structure was stable. Further validation factor analysis showed that the model fit was good, and the factor loadings of the subscale items ranged from 0.529 to 0.891, and the items were able to effectively measure the corresponding dimensions. The Chinese version of the HLS-14 is a reliable multidimensional HL measurement scale, and healthcare professionals can use it to accurately measure the HL levels of pregnant women with GDM.

#### 4.1.2 Applicability analysis of the HL-14

The Chinese version of the HLS-14 is a brief, clear scale, and we did not change the expression of the scale’s items as recommended by clinical GDM nursing experts, but only modified the guidance phrases of the scale’s dimensions to suit pregnant women with GDM. Previous studies have also rarely made any significant adjustments to the expression of the scale, and only made some differences in the scoring method (40, 49, 50), suggesting that the scale is applicable to different cultural and social backgrounds, population groups, and patients. With its clear, easy-to-understand semantic formulation, no ambiguous or difficult-to-understand statements, and short completion time, the scale can provide a quick, real-time assessment of a patient’s HL in a busy clinic setting. The assessment of maternal HL in previous studies has mostly used question-based items to assess their knowledge acquisition and utilization, which is biased toward functional HL, but in clinical decision-making and maternal and child health care, patients not only need to have sufficient functional HL to understand the information, but also need communicative and critical HL to acquire, evaluate and apply the information (51). The scale provides a multidimensional evaluation of the HL level of pregnant women with GDM, and its content is universal, does not involve the judgment of “right” or “wrong” of specific disease-related knowledge, but focuses on the subjective evaluation of the subject’s ability. It has been used in different countries and populations with proven reliability (15, 34, 38, 52, 53), and has been shown to have good reliability and validity in this study. Which makes it a high-quality assessment tool for evaluating the HL in the GDM population. In subsequent studies, the applicability of HLS-14 in Chinese maternity and other diverse populations can also be explored in favor of cross-sectional comparisons of HL levels.

### 4.2 The level and influence factors of HL in pregnant women with GDM

HL is the motivation, knowledge and ability of people to access, understand, evaluate and apply health information. It is assessed in a variety of ways, but all focus on the individual’s ability to access, process, and understand health information and services. In this study, 34.03% of pregnant women with GDM had adequate HL levels and 24.09% had limited HL levels, and the overall HL score was (3.26 ± 0.41), which is moderate and higher than the studies of Ousseine et al. (38) and Koster et al. (54). In Ousseine’s study, 66.8% of the participants were over 40 years and 45.8% had a history of cancer; in Koster’s study, the participants were all elective surgery patients with a mean age of 56.4 years. The race, age, and health status of the participants in both studies differed significantly from our study, which may partially explain the different levels of HL. In all dimensions, pregnant women with GDM had the lowest functional HL scores, followed by critical and communicative HL, which is inconsistent with the results of previous studies, in which Merker et al. (53) found that patients with neurofibromatosis had higher scores on the functional HL than on the communicative and critical HL; Ousseine’s survey of 2,342 Dutch HL found that communicative HL scored highest, followed by functional and critical HL (38); A survey among 225 patients undergoing elective surgery showed that the level of critical HL was much lower than functional and communicative HL (54).

In our study, the lowest scoring items all belonged to the functional HL dimension, which were “Takes a long time to read and understand”, “Find the font too small” and “Find the content too difficult to understand.” Given the overall high literacy level of pregnant women in this study (91.01% with college or higher education), they did not lack basic reading comprehension skills. The reason for this may be due to deficiencies in the form as well as the content of the health education materials provided by the medical institutions, such as excessive content, overly specialized presentation, and small font printing. It suggests that healthcare professionals should be more humane and personalized in their health education approach and methods, and give full consideration to the readability of health education materials.

The results of multivariate stepwise linear regression showed that family support, use of a pregnancy management diary, family income, pre-pregnancy BMI, time spent searching for health information, and searching for health information through medical information websites were the independent influences on HL in pregnant women with GDM. Guo S found that social support was the strongest predictor of communicative and critical HL (55); and a systematic evaluation by De Wit L, which qualitatively synthesized the results of 26 studies, also showed that social support was a key factor in improving critical HL among community-dwelling older adults, which promotes the ability to understand, judge, select, and apply health information (56). Social support systems, including peer support and family support, can alleviate patients’ psychological stress, effectively improve their psychological resilience and enhance their self-efficacy, which in turn promotes the maintenance of healthy self-management behaviors. Family support is an important part of social support, and a good family support system can provide life support, information support and emotional support for pregnant women with GDM, promote effective communication, and gain more support in accessing, understanding, and applying health information to facilitate their HL. In clinical work, healthcare professionals should pay attention to the positive role of family support on the level of HL, incorporate the family support system of pregnant women in the process of HL-promoting interventions, and fully mobilize their husbands and co-dependents for life support, emotional support, and informational support, such as providing comprehensive life care, regular heart-to-heart talks and joint participation in maternity education programs (57). Peer support is another important aspect of social support. Considering the affordability and convenience, seeking peer support through online communities is a good way, such as joining WeChat groups established by hospitals and GDM groups on online communication platforms. Where GDM pregnant women can seek and provide information support, sharing self-management tips and so on (58). The aims of all these support systems are to achieve the goals of effectively obtaining high-quality health information and optimizing health behaviors.

In our study, most of the pregnant women with GDM (93.50%) use a pregnancy management diary occasionally or frequently to record their daily diet, exercise, weight, blood glucose monitoring, and fetal movement. Pregnancy management diary is one of the common obstetrics self-management behavioral interventions for special maternity cases such as GDM. We found that pregnant women who kept a pregnancy management diary had higher HL levels, which may be due to the following reasons: on the one hand, pregnant women with GDM who kept a pregnancy management diary may pay more attention to their health management, their behaviors themselves are a manifestation of high HL levels; on the other hand, keeping a pregnancy management diary prompts pregnant women to make dynamic comparisons of their well-being, which is more conducive to facilitating the reflection on and optimization of their own health behaviors. Therefore, in the clinical practice of effectively improving HL in pregnant women with GDM, healthcare professionals should increase pregnant women’s attention to their own health behaviors, encourage pregnant women to record their health behaviors during pregnancy, promote reflection and improvement, and enhance the level of HL.

In addition, pre-pregnancy BMI is a negative influence on HL in pregnant women with GDM, and the higher the pre-pregnancy BMI, the lower the level of HL. As with HL, BMI is also an important indicator for evaluating disease severity, risk factor burden and quality of life, and it is a commonly used outcome indicator for the effect of lifestyle interventions, which to some extent can reflect people’s different lifestyles and health behaviors (59). A systematic review of HL and obesity-related problems in adults and children by Maria K found that HL had a determinative role in the management of BMI and ensuing health-related problems in both young and old people, individuals with high HL level were more likely to adopt healthier lifestyles and have a lower prevalence of obesity (60). It has also been found that adequate HL helps to promote the maintenance of an appropriate BMI over time in patients undergoing bariatric surgery (61). Pre-pregnancy BMI mainly reflects the pre-pregnancy lifestyle and health behaviors, which indirectly reflects their HL level. In addition, the weight management requirements of GDM pregnant women with higher BMI are more stringent than normal pregnant women (62), requiring extra maternal efforts, at which time HL becomes particularly important. Healthcare providers should place emphasis on pre-pregnancy BMI in pregnant women to recognize low HL in time.

Our study found that both income level and information access to health information were influences on HL in pregnancy with GDM, who with higher incomes, who spent more time search for health information, and who accessed information through healthcare information websites showed higher HL. BL Yong (63) used the Chinese National HL Questionnaire to survey 4,500 older adults in 44 nursing institutions, and similarly found that HL was closely related to health behaviors, and that income level and access to health information were independent influences on HL, differing in that the influences on HL in that study also included education level, occupation status, and age, whereas in our study, education level and occupation status were only correlated with HL in the univariate analysis. The strong correlation between income level, economic status and HL has also been confirmed in a number of studies, where people with financial difficulties are likely to report insufficient functional, communicative, and critical HL in the health care delivery system, economic deprivation is one of the main predictors of limited HL (64, 65). Therefore, healthcare providers should focus on economically disadvantaged pregnancies with GDM in healthcare services, improve health education methods and intervention strategies to ensure that health information is effectively accessed, correctly understood and applied.

In addition, with the development of the Internet, information dissemination has become fast and efficient, and the ways of dissemination are also widely diversified. The convenience of information acquisition makes people face massive information bombardment in everyday life, and browse information hurriedly has gradually become a habit, so how to obtain reliable information is a challenge for them. The identification of health information to make informed health decisions is a key component of critical HL (64), in our study, the majority (94.2%) of GDM pregnant women were proactive in accessing health information about pregnancy and the postpartum period, but only 59.5% of them judged the information were useful; Regarding the ways of obtaining information, the Internet has become the main way. This means that despite the variety of ways to access information, the quality of the information is still problematic, probably because the resources available on the Internet include not only those from specialized official institutions, but also many unofficial sources and even personal experiences, whose authenticity, reliability and professionalism are not guaranteed. We found that spending more time searching for information and accessing health information from healthcare websites predicted better HL levels, which may be due to that pregnant women who spend more time thinking about and understanding the information will be more in-depth compared to quick browsing, which is conducive to critically processing the information and achieving better HL levels. In addition, people with high HL are more inclined to obtain information from a variety of ways, and healthcare information websites are likely to be more professional and reliable in the provision of health information in favor of HL compared to short videos and Q&A platforms. For healthcare professionals, it is also crucial to assess and intervene in the ways and means of information access for pregnancies with GDM.

### 4.3 Limitations and future directions

There are several limitations. First, the study recruited participants from a tertiary teaching hospital in Chengdu, Sichuan Province, these samples may be representative of pregnant women in hospitals at this level only. Second, all information was obtained from questionnaires filled out by pregnant women themselves, recall bias may be present. Third, this study is a survey study and causal interpretation may be inadequate. Furthermore, considering the specific cultural and geographical setting of this study, for example, the special dietary culture of the southwestern region (a spicy, oil- and salt-heavy diet) is quite different from that of other regions of China. In addition, women in the southwest China generally have a higher social and family standing, and are more autonomous and self-reliant. This may lead to differences in health literacy and self-management behaviors among pregnant women with GDM compared to other regions. These particular cultural factors might affect the generalizability of the results. Moreover, this study only investigated a part of the influencing factors of health literacy due to the limitation of time, manpower, and funding, and may have ignored other important influencing factors, such as psychological factors, which resulted in a low degree of explanation of the regression model. We would conduct a more scientific design and use more reliable research methods in our future studies to fill these gaps. We also hope that further studies in other types of hospitals and in other regions of China with prospective cohort studies will yield more reliable results.

## 5 Conclusion

In this study, we investigated the level of HL in pregnant women with GDM through a cross-sectional survey study, explored the influencing factors based on the Integrated model of health literacy, and validated the applicability of the Chinese version of the HLS in pregnant women with GDM. The study showed that the Chinese version of the HLS is short, ideographically clear, and time-consuming to complete, and has good reliability and validity among pregnant women with GDM, making it a high-quality assessment tool for HL in the GDM population, which is favorable for the measurement of HL in pregnant women with GDM as well as for cross-sectional comparisons.

Moderate HL levels in pregnant women with GDM, with functional HL levels lower than communicative and critical HL. Healthcare providers should focus on GDM populations with low income and high pre-pregnancy BMI, fully mobilize their social support systems, provide reliable access to information, encourage all pregnant women with GDM to use pregnancy management diaries to record self-management, and ensure the effectiveness of health education.

## Data Availability

The original contributions presented in the study are included in the article/supplementary material, further inquiries can be directed to the corresponding authors.
